# Dose-dependent effects of selenium (Se)-enriched yeast containing nano-scale Se particles on Se bioavailability, rumen fermentation, hematological profile, and growth performance in female Thin-Tail sheep

**DOI:** 10.14202/vetworld.2026.3372-3384

**Published:** 2026-08-04

**Authors:** Dadik Pantaya, Merry Muspita Dyah Utami, Suci Wulandari, Hariadi Subagja, Muhammad Adhyatma, Agus Hadi Prayitno

**Affiliations:** 1 Animal Science Department, Politeknik Negeri Jember, Jl. Mastrip 164, Jember, Indonesia.

**Keywords:** animal performance, blood selenium, nano-selenium, rumen fermentation, selenium bioavailability, selenium-enriched yeast, Thin-Tail sheep, volatile fatty acids

## Abstract

**Background and Aim:**

Selenium (Se) deficiency remains a significant nutritional challenge in sheep production due to the limited bioavailability of inorganic Se sources and the low Se availability in many soils and forages. Se-enriched yeast containing nano-scale Se particles may provide a more bioavailable organic Se source while also influencing rumen fermentation and animal physiology. This study evaluated the dose-dependent effects of Se-enriched yeast containing nano-scale Se particles on Se bioavailability, rumen fermentation, hematological profile, and growth performance in female Thin-Tail sheep.

**Materials and Methods:**

Fifteen clinically healthy female Thin-Tail sheep (8 months old; 15 ± 3 kg) were randomly assigned to three dietary treatments in a completely randomized design (n = 5/group): basal diet without supplementation (P1), basal diet supplemented with 0.35 g Se-enriched yeast/head/day (P2), and basal diet supplemented with 0.70 g Se-enriched yeast/head/day (P3). The Se-enriched yeast contained 410 ppm Se and was characterized by nano-scale Se-associated particles. Feed intake, body weight, blood Se concentration, hematological variables, and rumen volatile fatty acid (VFA) profiles were evaluated. Data were analyzed using one-way analysis of variance, followed by Tukey’s multiple comparison test, with statistical significance set at p < 0.05.

**Results:**

Particle size analysis confirmed Se-associated particles within the nano-scale range (149.2–191.1 nm). Se-enriched yeast supplementation significantly increased blood Se concentration in a dose-dependent manner (p = 0.001), with mean concentrations increasing from 98.70 ppb in P1 to 156.50 ppb and 206.56 ppb in P2 and P3, respectively. Iso-valerate and total branched-chain iso-acids in rumen fluid also increased significantly in supplemented sheep (p < 0.05), indicating enhanced ruminal nitrogen metabolism and microbial activity. However, total VFA concentration, major VFA proportions, body weight gain, and most hematological variables were not significantly affected (p > 0.05). Feed intake differed only during the first experimental week and remained comparable thereafter.

**Conclusion:**

Dietary supplementation with Se-enriched yeast containing nano-scale Se particles effectively improved systemic Se status in a dose-dependent manner and selectively enhanced ruminal branched-chain VFA production without adversely affecting hematological homeostasis or growth performance. Supplementation at 0.35–0.70 g/head/day appears to be a safe and highly bioavailable strategy for improving Se nutrition in female Thin-Tail sheep and may contribute to improved rumen microbial function under tropical production conditions.

## INTRODUCTION

Selenium (Se) is an essential trace mineral that plays a critical role in antioxidant defense, immune regulation, and metabolic function in ruminants, including sheep. Se is an integral component of selenoproteins, particularly glutathione peroxidase, which protects cells against oxidative damage and maintains normal physiological functions [1, 2]. In sheep, Se deficiency has been associated with reduced growth performance, impaired immune function, compromised reproductive efficiency, and an increased risk of metabolic disorders [3, 4]. In many regions, particularly tropical and subtropical areas, Se concentrations in soils and forage are insufficient, resulting in inadequate dietary Se intake by grazing sheep [5, 6]. This situation is also relevant in Indonesia, where tropical environmental conditions can influence Se availability in soils and forage resources, emphasizing the need for highly bioavailable Se sources to support sustainable ruminant production [7]. Consequently, Se supplementation has become an important nutritional strategy to improve animal health and productivity by enhancing antioxidant defense, immune modulation, and metabolic regulation. Previous studies have demonstrated that organic Se supplementation can improve rumen fermentation, nutrient digestibility, microbial stability, antioxidant status, and physiological performance in ruminants [8]. However, the biological effectiveness of Se supplementation is highly dependent on its chemical form. Inorganic Se sources, such as sodium selenite, generally exhibit lower bioavailability and possess a narrower margin between nutritional adequacy and toxicity than organic Se sources [6].

Se-enriched yeast is considered one of the most bioavailable organic Se sources available for livestock nutrition. During microbial fermentation, yeast incorporates inorganic Se into organic Se compounds, predominantly selenomethionine, which can be efficiently absorbed through amino acid transport systems [9]. In addition, the fermentation process may generate Se-associated particles within the nano-scale range in the yeast biomass, potentially enhancing intestinal absorption, tissue retention, and biological utilization of Se [10]. Beyond serving as a Se carrier, yeast also contains bioactive compounds, including amino acids, peptides, B-complex vitamins, β-glucans, and mannan oligosaccharides, which contribute to improved gut health, nutrient utilization, rumen microbial activity, and metabolic efficiency in ruminants. Previous studies have demonstrated that yeast supplementation improves microbial balance, nutrient digestibility, and physiological performance in sheep [11, 12]. Nevertheless, the biological effects of Se-enriched yeast containing nano-scale Se-associated particles on Se bioavailability and rumen fermentation characteristics remain insufficiently understood.

Although numerous studies have investigated conventional organic Se supplementation in ruminants, most have primarily focused on antioxidant status, growth performance, immune responses, or Se deposition in tissues. Limited information is available regarding Se-enriched yeast containing naturally occurring nano-scale Se-associated particles produced during fermentation, particularly under tropical production systems. Furthermore, few studies have simultaneously evaluated its effects on systemic Se bioavailability, rumen fermentation characteristics, hematological profile, and productive performance in sheep. In particular, the influence of this Se source on branched-chain volatile fatty acids (VFAs), including iso-butyrate and iso-valerate, which serve as indicators of ruminal nitrogen metabolism and microbial activity, has received little attention. Consequently, the nutritional and physiological potential of locally produced nano-characterized Se-enriched yeast in Indonesian Thin-Tail sheep has not been comprehensively investigated.

Therefore, this study aimed to evaluate the dose-dependent effects of an *in vitro*-produced, nano-characterized Se-enriched yeast on Se bioavailability, rumen fermentation characteristics, hematological profile, and growth performance in female Indonesian Thin-Tail sheep. Particular emphasis was placed on determining changes in blood Se concentration and ruminal branched-chain VFAs, including iso-butyrate and iso-valerate, as indicators of Se utilization and rumen microbial metabolism under tropical feeding conditions. It was hypothesized that dietary supplementation with Se-enriched yeast containing nano-scale Se-associated particles would enhance systemic Se status and beneficially modulate ruminal branched-chain VFA production without adversely affecting hematological homeostasis or growth performance.

## MATERIALS AND METHODS

### Ethical approval

All experimental procedures involving animals were reviewed and approved by the Institutional Animal Care and Use Committee (IACUC) of Politeknik Negeri Jember, Jember, Indonesia (Approval No**. **0240/PL17.4/ PG/2026). The study was conducted in accordance with the institutional guidelines for the care and use of experimental animals, the applicable national regulations governing animal research in Indonesia, and internationally accepted principles for the ethical use of animals in scientific investigations.

Before initiation of the experiment, all sheep underwent a comprehensive clinical examination by qualified veterinarians to confirm that they were clinically healthy, free from infectious diseases, and suitable for inclusion in the study. Throughout the experimental period, animals were maintained under standard commercial husbandry conditions with unrestricted access to clean drinking water and nutritionally balanced diets formulated to meet their physiological requirements. Routine health monitoring was performed daily, and all animals received appropriate preventive veterinary care, including deworming before the commencement of the feeding trial.

All experimental procedures, including oral administration of Se-enriched yeast, jugular venous blood collection, and rumen fluid sampling using an oral stomach tube, were performed by trained personnel using standardized veterinary techniques under strict hygienic and aseptic conditions. Animal restraint was limited to the minimum duration necessary to safely complete each procedure, and every effort was made to minimize pain, stress, and discomfort, as well as unnecessary handling, throughout the study. No surgical procedures, invasive interventions requiring anesthesia, or euthanasia were performed as part of the experimental protocol.

The study complied with the principles of the 3Rs (Replacement, Reduction, and Refinement) by using the minimum number of animals required to achieve scientifically valid results, refining all procedures to minimize animal distress, and ensuring that no unnecessary procedures were performed. Animals showing any signs of illness or abnormal clinical conditions would have received immediate veterinary attention and, if necessary, been withdrawn from the experiment to safeguard their welfare.

### Study period and location

The animal experiment was conducted from January to February 2026 at a commercial sheep farm in Hambalang, Citeureup, Bogor Regency, West Java, Indonesia. The farm is located in a highland area at an altitude of approximately 630 m above sea level, representing typical tropical environmental conditions for sheep production.

### Study design

The study was conducted using a completely randomized design (CRD). Fifteen clinically healthy female Thin-Tail sheep with a uniform age (8 months) and average body weight of 15 ± 3 kg were randomly assigned to three dietary treatments (n = 5 per treatment). Initial body weight was balanced among treatment groups to minimize experimental variation. Animals were housed individually in wooden pens (100 × 125 cm) with free access to feed and clean drinking water. The experiment consisted of a 14-day adaptation period followed by a 28-day feeding trial.

### Organic Se-yeast preparation

The *Saccharomyces cerevisiae* strain used in this study was obtained from the Feed Technology Laboratory culture collection at Politeknik Negeri Jember, Jember, Indonesia. Se-enriched yeast was produced by fermentation in a growth medium containing peptone, sucrose, and molasses, along with a mineral mixture of magnesium sulfate, zinc sulfate, ferric chloride, boric acid, copper sulfate, and sodium chloride. The medium was supplemented with sodium selenite (Na₂SeO₃·5H₂O) obtained from a local chemical supplier (Jakarta, Indonesia). The fermentation procedure followed previously published methods [13, 14]. Fermentation was performed at 30°C for 48 h under acidic conditions (pH 5) [15]. Following fermentation, the Se-enriched yeast biomass was dried and ground to pass through a 1-mm sieve before dietary supplementation.

### Nano-scale Se-yeast particle characterization

Particle size analysis (PSA) was performed before dietary supplementation to verify the presence of nano-scale Se-associated particles in the Se-enriched yeast. The average particle diameter, particle size distribution, and polydispersity index (PDI) were determined to characterize particle size uniformity and dispersion. Measurements were performed at 25°C using ethanol as the dispersing medium, with a refractive index of 1.36 and viscosity of 1.1015 cP.

Particle morphology was examined using scanning electron microscopy (SEM). Particle size was determined using a Delsa™ Nano analyzer (Beckman Colter, Brea, CA, USA). Briefly, 1000 ppm Se-enriched yeast was dispersed in ethanol and ultrasonicated for 5–10 min to ensure a homogeneous particle dispersion prior to measurement.

The Se concentration of the product was determined by atomic absorption spectrophotometry (AAS) according to a previously described method [16]. Analytical accuracy was verified using Randox Control Sera (Randox Laboratories Ltd., Antrim, UK). These analyses were performed using standard nanoparticle characteri-zation techniques to confirm the classification of the Se-enriched yeast as a nano-scale Se source.

### Dietary treatments

All sheep received a basal diet formulated to satisfy the nutrient requirements of growing sheep (Table 1). The nutrient composition, including dry matter (DM), crude protein (CP), crude fiber (CF), and ash, was determined according to AOAC procedures [17]. DM was determined by oven drying, CP by the Kjeldahl method, CF by sequential acid-alkali digestion, and ash by furnace combustion.

Throughout the 28-day experimental period, sheep were fed a forage-to-concentrate ratio of 70:30. Before the experiment, all animals were clinically examined to ensure that they were healthy, free of apparent infectious diseases, and fully adapted to the experimental environment. Animals were routinely dewormed and maintained under standard farm health management practices throughout the study.

Se-enriched yeast was used as the dietary source of Se. The experimental treatments were:

1. **P1:** Basal diet without Se-enriched yeast supplementation (control).

2. **P2:** Basal diet supplemented with 0.35 g Se-enriched yeast/head/day.

3. **P3:** Basal diet supplemented with 0.70 g Se-enriched yeast/head/day.

The control diet contained only naturally occurring Se derived from the basal feed ingredients and was included to evaluate the comparative bioavailability and physiological effects of supplemental organic Se under identical feeding conditions.

Experimental diets were offered daily, and all animals had unrestricted access to clean drinking water. Supplementation levels were selected based on previous studies of organic Se supplementation in ruminants and adjusted to the product's Se concentration (410 ppm Se). These supplementation rates supplied approximately 0.14 mg Se/head/day in P2 and 0.29 mg Se/head/day in P3, in addition to the naturally occurring dietary Se. Se-enriched yeast was weighed daily according to treatment, encapsulated in gelatine capsules, and orally administered to individual sheep before the morning feeding to ensure complete and accurate intake.

Animal performance was assessed by measuring feed intake and body weight gain. Feed intake was recorded daily as the difference between feed offered and feed refused. Body weight was measured at the beginning and end of the experimental period, and body weight gain was subsequently calculated.

**Table 1:** Ingredients and nutrient composition of the basal diet.

**Item**	**Forage**	**Concentrate**
Ingredients (% of diet DM)		
Native field grass	100.0	–
Palm meal	–	22.5
Cassava meal	–	25.0
Coffee pod meal	–	10.0
Ketchup meal	–	8.5
Rice bran meal	–	23.0
Wheat pollard	–	10.0
Mineral and vitamin mixture¹	–	1.0
Nutrient composition (% of DM)		
DM	20.0	89.8
CP	7.5	9.3
CF	29.0	26.5
Ash	12.0	8.2
TDN	55.0	50.2

¹Mineral composition: P, 0.25%; Ca, 2.0%; Mg, 0.45%; and Na, 0.35%. Trace element composition: Cu, 15 mg/kg. Vitamin composition: vitamin A, 6000 IU/kg; vitamin D₃, 1250 IU/kg; and vitamin E, 10 mg/kg. DM: Dry matter; CP: Crude protein; CF: Crude fiber; TDN: Total digestible nutrients. The Se concentration of the basal diet was not directly analyzed.

### Blood sampling, hematological analysis, and blood Se determination

Blood samples were collected from the jugular vein of each sheep before the morning feeding at the end of the experimental period using sterile procedures. A portion of each sample was used immediately for hematological analysis to assess the animals' physiological status.

Approximately 5 mL of blood was collected into ethylenediaminetetraacetic acid tubes for hematological analysis using an automated veterinary hematology analyzer (Mindray BC-2800Vet; Shenzhen Mindray Bio-Medical Electronics Co., Ltd., Shenzhen, China) according to the manufacturer's instructions. Blood samples intended for Se analysis were collected into plain tubes, centrifuged at 3,000 rpm (approximately 1.500 × *g*) for 15 min to obtain serum, and stored at −20°C until analysis.

Blood Se concentration was determined as an indicator of Se absorption and systemic Se status. Serum samples were subjected to wet digestion and analyzed using hydride-generation AAS (PerkinElmer Analyst Series; PerkinElmer Inc., Waltham, MA, USA) at a wavelength of 196 nm. Se standard solutions were used for instrument calibration, and analytical accuracy was verified using certified control sera. The analytical recovery rate ranged from 95% to 103%, with an intra-assay coefficient of variation of <5%.

### Rumen fluid sampling and VFA analysis

Rumen fluid samples were collected using an oral stomach tube connected to a vacuum pump immediately before feeding (0 h) and 4 h after feeding. The initial portion of rumen fluid was discarded to minimize saliva contamination. Approximately 50 mL of rumen fluid was filtered through four layers of cheesecloth and transferred into polypropylene tubes.

For VFA analysis, approximately 0.8 mL of filtered rumen fluid was mixed with 0.5 mL of a stabilizing solution containing crotonic acid (4 g/L) and metaphosphoric acid (20 g/L) dissolved in 0.5 N hydrochloric acid [18]. The mixture was homogenized and immediately stored at −20°C until analysis.

VFA concentrations were determined by gas chromatography equipped with a flame ionization detector and a capillary column suitable for short-chain fatty acid separation. Injector and detector temperatures were maintained at 220°C and 250°C, respectively, while the column temperature was programmed according to standard VFA analytical procedures. Analytical validation demonstrated satisfactory peak separation and repeatability for acetate, propionate, butyrate, iso-butyrate, valerate, and iso-valerate.

### Statistical analysis

All data were analyzed using one-way analysis of variance (ANOVA) under a CRD using Minitab® Version 13 (Minitab Inc., State College, PA, USA). The analyzed variables included feed intake, body weight gain, blood Se concentration, hematological variables (glucose, erythrocytes, hemoglobin, leukocytes, hematocrit, mean corpuscular volume (MCV), mean corpuscular hemoglobin (MCH), mean corpuscular hemoglobin concentrations (MCHC), neutrophils, lymphocytes, monocytes, and platelets), and rumen fermentation characteristics, including individual and total VFA concentrations.

Each sheep was considered the experimental unit. Data normality and homogeneity of variance were assessed using the Shapiro-Wilk and Levene's tests, respectively, before conducting ANOVA. Blood Se concentration, hematological variables, and body weight gain were analyzed using one-way ANOVA. Feed intake, body weight, and rumen fermentation variables were analyzed separately for each sampling period.

The statistical model was:

Yᵢⱼ = μ + Tᵢ + εᵢⱼ

where Yᵢⱼ is the observed value of the dependent variable, μ is the overall mean, Tᵢ is the fixed effect of dietary treatment (i = 1, 2, and 3), and εᵢⱼ is the random error.

When significant treatment effects were detected, means were separated using Tukey's multiple comparison test. Statistical significance was declared at p < 0.05, whereas 0.05 ≤ p < 0.10 was considered indicative of a statistical trend.

## RESULTS AND DISCUSSION

### Particle size characteristics of Se-enriched yeast

**Figure 1:**
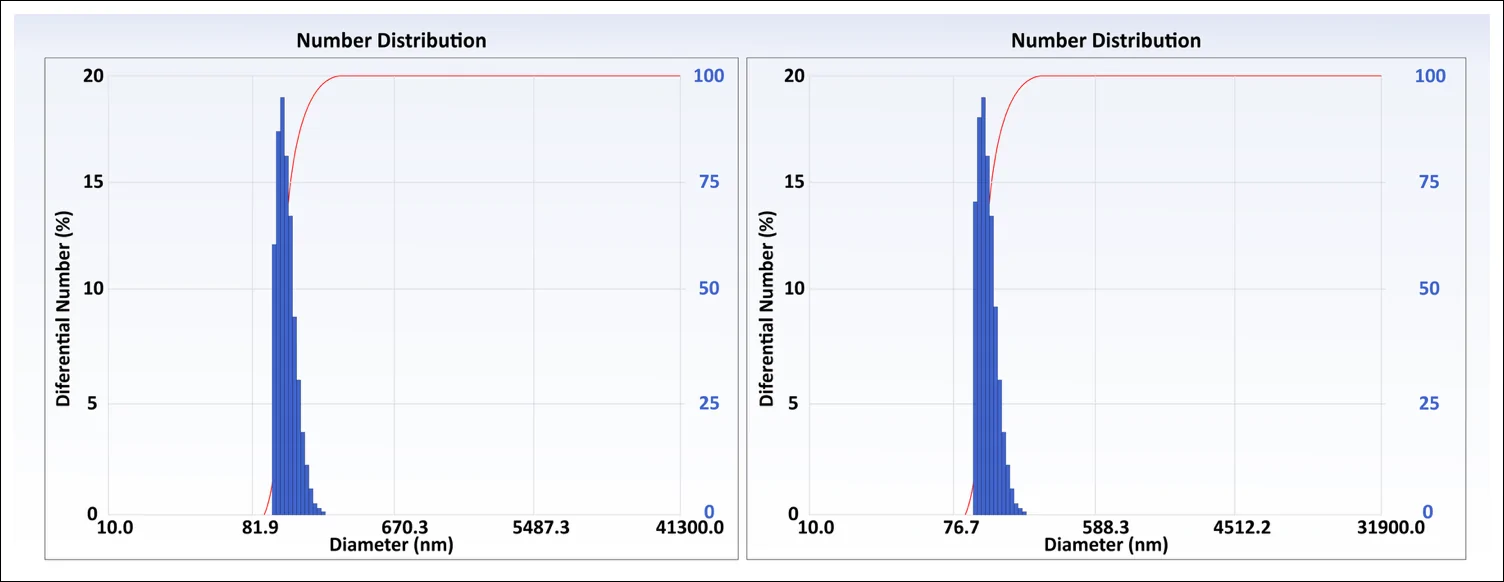
Particle size distribution of selenium-enriched yeast showing dominant particle size peaks at 149.2 and 191.1 nm and PDI values ranging from 0.554 to 0.654.

PSA confirmed the presence of Se-associated particles within the nanometer range (Figure 1). The dominant particle size peaks were observed at 149.2 and 191.1 nm for Se-enriched yeast samples 2 and 3, respectively. Secondary peaks at particle sizes >14 µm indicated some degree of particle aggregation. The PDI values ranged from 0.554 to 0.654, indicating a moderately heterogeneous particle-size distribution. Nevertheless, the dominant peaks remained within the nano-scale range, confirming the successful formation of nano-scale Se-associated particles during fermentation [19, 20].

The nano-scale particle sizes identified in the present study are nutritionally relevant because smaller Se particles provide a relatively large surface area, which may facilitate intestinal absorption and biological utilization compared with conventional inorganic Se sources [21, 22]. Previous studies have reported that nano-Se may exhibit greater bioavailability and metabolic efficiency, together with lower toxicity, than inorganic Se sources [23, 24].

Although the PDI values indicated moderate heterogeneity and some particle aggregation, the dominant particle-size peaks confirmed the presence of nano-scale Se-associated particles in the Se-enriched yeast product [25]. These physicochemical characteristics may facilitate the transport, cellular uptake, and incorporation of Se into metabolic pathways, thereby improving systemic Se status following supplementation [20, 26]. However, because Se speciation and the biological contribution of the nano-scale fraction were not directly evaluated, the improved Se status cannot be attributed exclusively to the nano-scale particles.

**Figure 2:**
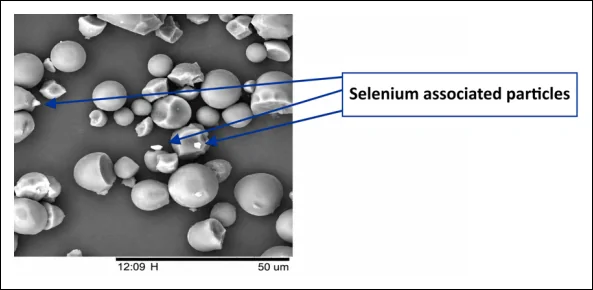
Scanning electron microscopic image of Se-enriched yeast showing particle morphology and aggregated Se-associated structures within the yeast matrix.

Overall, the PSA findings in Figure 1 indicate that the fermented Se-enriched yeast contained Se-associated particles predominantly in the 149.2–191.1 nm range. These particle characteristics may partly explain the enhanced systemic Se concentrations observed in supplemented sheep [27, 28]. Figure 2. shows the scanning electron microscopic image of the Se-enriched yeast, illustrating the morphology and aggregated Se-associated particles.

### Growth performance

Feed intake was significantly higher in P1 than in P2 and P3 during the first week of the experimental period (p < 0.05; Table 2). However, feed intake did not differ significantly among treatments during weeks 2–4 (p > 0.05), indicating that Se-enriched yeast supplementation had no persistent effect on voluntary feed intake.

**Table 2:** Weekly feed intake of female Thin-Tail sheep (n = 5 per treatment).

**Time**	**P1**	**P2**	**P3**	**SEM**	**p-value**
Week 1	11.029ᵃ	9.212ᵇ	9.765ᵇ	0.630	0.031
Week 2	12.068	11.423	10.610	0.632	0.079
Week 3	10.207	10.677	10.061	0.100	0.740
Week 4	10.180	10.133	9.235	0.742	0.280
Weeks 1–4	43.848	41.444	36.672	1.788	0.102

Values are expressed as kg/head/week. P1: Basal diet without Se-enriched yeast supplementation; P2: Basal diet supplemented with 0.35 g Se-enriched yeast/head/day; P3: Basal diet supplemented with 0.70 g Se-enriched yeast/head/day; SEM: Standard error of the mean. Values with different superscripts within the same row differ significantly (p < 0.05).

The initial difference in feed intake may reflect short-term adaptation to supplementation rather than a sustained effect on appetite or diet palatability. Similar findings have been reported in sheep, in which mineral supplementation did not consistently increase feed intake after animals had adapted to the experimental diets [11, 29]. The absence of treatment-related differences after the first week indicates that supplementation at either dose did not adversely affect feed acceptance.

Body weight and body weight gain did not differ significantly among treatments during the 4-week experimental period (p > 0.05; Table 3). Mean body weight followed a comparable pattern across the three groups, indicating that Se-enriched yeast supplementation did not promote measurable growth under the conditions of this study. The relatively short supplementation period may have limited the ability to detect gradual treatment-related changes in growth. Comparable findings have been reported by Liu *et al.* [12], who observed physiological benefits from supplementation without a corresponding increase in body weight.

**Table 3:** Body weight and body weight gain of female Thin-Tail sheep (n = 5 per treatment).

**Parameter**	**P1**	**P2**	**P3**	**SEM**	**p-value**
Body weight at week 1 (kg)	16.27	13.83	13.60	1.39	0.40
Body weight at week 4 (kg)	20.37	21.93	20.00	5.98	0.76
Body weight gain during weeks 1–4 (kg)	4.10	8.10	6.40	2.69	0.48

P1: Basal diet without Se-enriched yeast supplementation; P2: Basal diet supplemented with 0.35 g Se-enriched yeast/head/day; P3: Basal diet supplemented with 0.70 g Se-enriched yeast/head/day; SEM: Standard error of the mean.

The absence of a significant growth response suggests that Se-enriched yeast may primarily support metabolic regulation and physiological homeostasis rather than directly promote growth. Se has a well-established role in antioxidant defense, immune regulation, and metabolic balance, but supplementation does not necessarily increase growth when the basal diet already provides sufficient nutrients for normal development [30].

### Hematological profile

Most hematological variables, including erythrocyte count, hemoglobin concentration, leukocyte count, hematocrit, and differential leukocyte counts, were not significantly affected by Se-enriched yeast supplementation (p > 0.05; Table 4). These findings indicate that supplementation at 0.35 and 0.70 g/head/day did not produce detectable adverse effects on hematological homeostasis.

The maintenance of hematological values across treatments suggests that the administered supplementation levels were physiologically well tolerated during the 28-day experimental period. Organic Se supplementation may support antioxidant balance and immune-cell function without necessarily altering routine hematological indices in clinically healthy animals [31]. Nevertheless, the absence of measurements of antioxidant enzymes or oxidative-stress biomarkers limits the interpretation of the functional effects of the improved Se status.

**Table 4:** Blood profile of female Thin-Tail sheep (n = 5 per treatment).

**Parameter**	**P1**	**P2**	**P3**	**SEM**	**p-value**
Glucose (mg/dL)	51.00	52.00	50.67	3.92	0.91
HGB (g/dL)	9.47	9.93	7.60	1.68	0.27
RBC (×10⁶/µL)	6.20	6.53	5.74	0.69	0.43
HCT (%)	25.73	28.17	23.57	3.64	0.36
MCV (fL)	41.47	42.87	41.03	1.21	0.23
MCH (pg)	15.27	15.07	13.30	1.64	0.33
MCHC (g/dL)	36.87	35.03	32.43	3.63	0.38
WBC (×10³/µL)	13.39	10.56	9.50	2.83	0.30
Neutrophils (%)	0.45	0.37	0.50	0.08	0.21
Lymphocytes (%)	78.40	97.50	97.93	19.74	0.43
Monocytes (%)	1.63	2.13	1.57	0.62	0.51
PLT (/µL)	1,116,333	807,000	702,000	386.120	0.44

P1: Basal diet without Se-enriched yeast supplementation; P2: Basal diet supplemented with 0.35 g Se-enriched yeast/head/day; P3: Basal diet supplemented with 0.70 g Se-enriched yeast/head/day; HGB: Hemoglobin; RBC: Red blood cell count; HCT: Hematocrit; MCV: Mean corpuscular volume; MCH: Mean corpuscular hemoglobin; MCHC: Mean corpuscular hemoglobin concentration; WBC: White blood cell count; PLT: Platelet count; SEM: Standard error of the mean.

### Blood Se concentration

Dietary Se-enriched yeast supplementation significantly increased blood Se concentration in a dose-dependent manner (p = 0.001; Table 5). Blood Se concentration increased from 98.70 ppb in P1 to 156.50 ppb in P2 and 206.56 ppb in P3. Therefore, sheep receiving 0.35 g Se-enriched yeast/head/day had significantly higher blood Se concentrations than the control sheep, whereas the highest concentration was recorded in sheep receiving 0.70 g/head/day.

**Table 5:** Blood Se concentrations in female Thin-Tail sheep (n = 5 per treatment).

**Parameter**	**P1**	**P2**	**P3**	**SEM**	**p-value**
Blood Se concentration (ppb)	98.70ᵃ	156.50ᵇ	206.56ᶜ	14.26	0.001

P1: Basal diet without Se-enriched yeast supplementation; P2: Basal diet supplemented with 0.35 g Se-enriched yeast/head/day; P3: Basal diet supplemented with 0.70 g Se-enriched yeast/head/day; ppb: Parts per billion; SEM: Standard error of the mean. Values with different superscripts within the same row differ significantly (p < 0.05).

Although Se was detected in the blood of control sheep, its concentration was significantly lower than those in both supplemented groups. The Se detected in P1 was presumably derived from naturally occurring Se in the basal feed ingredients. However, the lower concentration in this group indicates that the basal dietary supply did not achieve a systemic Se status comparable to that produced by supplementation.

The progressive increase in blood Se concentration with increasing supplementation dose demonstrates the effective absorption and systemic availability of Se supplied through Se-enriched yeast. Comparable dose-responsive increases in circulating Se have been reported in ruminants supplemented with organic Se sources [32]. Se-enriched yeast contains organic Se compounds, predominantly selenomethionine, which can be absorbed through amino acid transport pathways and incorporated into body proteins more efficiently than many inorganic Se forms [32, 33].

The improved Se status observed in P2 and P3 was therefore primarily attributable to the organic Se supplied by the Se-enriched yeast. The nano-scale Se-associated particles may also have contributed through their relatively large surface area and potential for enhanced cellular uptake [34]. However, this interpretation should be approached with caution because the relative contributions of selenomethionine, other organic Se compounds, and nano-scale Se fractions were not independently determined.

### Comparison of blood Se concentrations with previous studies

Blood Se concentrations reached 156.50 ppb in P2 and 206.56 ppb in P3. These concentrations were within the reported physiological range of approximately 100–300 ppb in sheep, although reference values may vary with dietary Se source, supplementation level, physiological status, analytical method, and geographical conditions [35, 36].

Orzechowski *et al.* [37] reported blood Se concentrations of approximately 140–180 ppb in sheep supplemented with an organic Se source, which are comparable to the concentration recorded in P2. Similarly, Sun *et al.* [38] documented blood Se concentrations exceeding 200 ppb in ruminants receiving higher levels of Se-enriched yeast, consistent with the concentration measured in P3.

The higher blood Se concentration in P3 than in P2 confirms a clear dose-dependent response. Organic Se, particularly selenomethionine, is efficiently absorbed in the small intestine and can be incorporated nonspecifically into body proteins in place of methionine, thereby enhancing Se retention in blood and tissues [11, 39]. The nano-scale characteristics of the Se-associated particles produced during yeast fermentation may further improve biological utilization by increasing particle surface area and cellular uptake efficiency [40, 41].

Overall, supplementation with 0.35–0.70 g Se-enriched yeast/head/day effectively improved systemic Se status without producing detectable hematological abnormalities. However, because the basal diet Se concentration and tissue Se deposition were not measured, the adequacy and long-term safety of these supplementation levels require further evaluation.

### Rumen VFA profile

The rumen VFA profile before feeding and 4 h after feeding is presented in Table 6. Before feeding, acetate, propionate, butyrate, valerate, and total VFA concentrations did not differ significantly among treatments (p > 0.05). Total VFA concentrations ranged from approximately 45 to 53 mM, indicating that Se-enriched yeast supplementation did not substantially alter basal carbohydrate fermentation.

In contrast, Se-enriched yeast supplementation significantly increased ruminal iso-valerate concentration and the total proportion of branched-chain iso-acids before feeding (p < 0.05). Iso-valerate is principally produced through the deamination of branched-chain amino acids, particularly leucine [42]. Branched-chain VFAs provide essential carbon skeletons and growth factors for several cellulolytic rumen bacteria and contribute to microbial protein synthesis, membrane-lipid formation, and cellular metabolism [12, 42]. Thus, the increase in iso-valerate may indicate enhanced branched-chain amino acid metabolism and greater availability of growth factors for fiber-degrading microorganisms.

**Table 6:** Ruminal VFA profile of female Thin-Tail sheep before and 4 h after feeding (n = 5 per treatment).

**Parameter**	**P1**	**P2**	**P3**	**SEM**	**p-value**
Before feeding (0 h)					
Total VFA (mM)	53.32	45.34	45.44	18.67	0.811
Acetate (C2; %)	77.36	77.49	76.04	2.37	0.716
Propionate (C3; %)	13.53	13.82	14.78	1.55	0.581
Iso-butyrate (iC4; %)	0.72	0.79	1.10	0.20	0.090
Butyrate (nC4; %)	7.40	6.90	6.50	1.49	0.737
Iso-valerate (iC5; %)	0.52ᵃ	0.72ᵃ	1.19ᵇ	0.13	0.001
Valerate (nC5; %)	0.48	0.32	0.39	0.21	0.616
Total iso-acids (%)	1.43ᵃ	1.47ᵃ	2.28ᵇ	0.31	0.008
Acetate-to-propionate ratio	5.77	5.68	5.18	0.74	0.582
4 h after feeding					
Total VFA (mM)	54.40	80.34	80.32	22.01	0.256
Acetate (C2; %)	60.09	56.99	57.35	8.96	0.881
Propionate (C3; %)	14.55	15.02	15.25	1.88	0.880
Iso-butyrate (iC4; %)	2.10	5.64	5.30	2.55	0.193
Butyrate (nC4; %)	15.80	17.14	17.72	6.84	0.930
Iso-valerate (iC5; %)	2.50	3.74	3.81	1.04	0.234
Valerate (nC5; %)	4.97	1.46	1.47	2.57	0.178
Total iso-acids (%)	4.59	9.38	9.11	3.05	0.128
Acetate-to-propionate ratio	3.76	3.80	3.76	0.76	0.695

P1: Basal diet without Se-enriched yeast supplementation; P2: Basal diet supplemented with 0.35 g Se-enriched yeast/head/day; P3: Basal diet supplemented with 0.70 g Se-enriched yeast/head/day; VFA: Volatile fatty acid; C2: Acetate; C3: Propionate; iC4: Iso-butyrate; nC4: Butyrate; iC5: Iso-valerate; nC5: Valerate; SEM: Standard error of the mean. Values with different superscripts within the same row differ significantly (p < 0.05).

These findings suggest that Se-enriched yeast selectively influenced ruminal nitrogen-related fermentation rather than overall carbohydrate fermentation. Previous studies have associated Se-enriched yeast supplementation with improved ruminal redox balance, stabilization of microbial ecology, maintenance of microbial viability, and modulation of ruminal enzyme activity [21, 43]. Nevertheless, because rumen microbial populations and enzyme activities were not measured directly, the proposed microbial mechanisms remain inferential.

At 4 h after feeding, individual and total VFA concentrations did not differ significantly among treatments (p > 0.05). Total VFA concentration was approximately 54 mM in P1 and numerically approached 80 mM in P2 and P3, but the differences were not statistically significant. The post-feeding increases reflected normal fermentation following feed intake and indicated that Se-enriched yeast supplementation did not disrupt the stability of postprandial rumen fermentation [44].

Overall, Se-enriched yeast supplementation selectively enhanced iso-acid production without significantly affecting total VFA concentration or the proportions of major VFAs before or after feeding. Similar observations have been reported in studies in which organic Se modulated microbial activity and nitrogen-related fermentation pathways without markedly altering total VFA production [45, 46]. This selective response may support microbial efficiency while maintaining overall rumen fermentation stability.

### Novel contributions and practical implications

The dose-dependent increase in blood Se concentration observed in this study is consistent with previous evidence demonstrating the high biological availability of Se-enriched yeast and nano-Se sources in ruminants [32, 45]. However, the present study extends these findings by demonstrating a concurrent increase in ruminal iso-valerate production, suggesting a potential relationship between improved Se status and ruminal branched-chain amino acid metabolism.

Another strength of this study is the evaluation of locally produced Se-enriched yeast containing nano-scale Se-associated particles in female Thin-Tail sheep maintained under tropical highland production conditions in Indonesia. The results indicate that supplementation at 0.35–0.70 g/head/day may be a practical strategy to improve systemic Se status without adversely affecting feed intake, growth performance, or hematological homeostasis. The increase in ruminal iso-acid production also suggests a potential benefit for microbial nitrogen metabolism. However, this proposed benefit requires confirmation through direct analyses of microbial populations, fiber digestibility, microbial protein synthesis, and ruminal enzyme activity.

### Study limitations and future research

This study has several limitations. First, the relatively small sample size and a 28-day supplementation period may have limited the statistical power to detect modest or long-term effects on growth, hematological characteristics, and rumen fermentation. Second, only female Indonesian Thin-Tail sheep were included; therefore, the findings may not be directly applicable to males, other age groups, animals with different physiology, or other sheep breeds.

Third, antioxidant biomarkers, including glutathione peroxidase, superoxide dismutase, and catalase, were not measured. Consequently, the study could not directly determine whether the increased blood Se concentration enhanced Se-dependent antioxidant activity. Tissue Se deposition, indicators of Se toxicity, reproductive responses, and long-term safety outcomes were also not evaluated.

Fourth, the Se concentration of the basal diet was not directly determined. Therefore, total dietary Se intake could not be calculated, and only the supplemental Se supplied through Se-enriched yeast was quantified. In addition, Se speciation analysis was not performed; thus, the relative proportions and biological contributions of selenomethionine, other organic Se compounds, and nano-scale Se fractions remain unknown. The moderately high PDI values and evidence of particle aggregation also indicate that further optimization of product uniformity may be required.

Future studies should include larger animal populations, longer supplementation periods, and direct measurement of total dietary and tissue Se concentrations. Comprehensive assessments of antioxidant status, toxicity indicators, nutrient digestibility, microbial protein synthesis, rumen microbial composition, and productive and reproductive performance are also warranted. Detailed Se speciation and nanoparticle characterization would help clarify the relative contribution of each Se fraction to systemic bioavailability and rumen fermentation responses.**Top of Form**

### Bottom of Form

## CONCLUSION

Supplementation with Se-enriched yeast containing nano-scale Se-associated particles effectively improved systemic Se status in female Thin-Tail sheep under tropical highland conditions. Blood Se concentration increased in a clear dose-dependent manner, from 98.70 ppb in the control group to 156.50 and 206.56 ppb in sheep supplemented with 0.35 and 0.70 g Se-enriched yeast/head/day, respectively. Se-enriched yeast supplementation also increased ruminal iso-valerate and branched-chain iso-acid production before feeding, suggesting a selective effect on ruminal branched-chain amino acid metabolism and microbial activity. In contrast, supplementation did not significantly alter body weight, body weight gain, most hematological variables, total VFA concentration, or the proportions of major VFAs, indicating that both supplementation levels were well tolerated during the 28-day feeding period.

A major strength of this study was the simultaneous evaluation of particle size characteristics, blood Se status, growth performance, hematological homeostasis, and rumen fermentation responses. Confirmation of dominant Se-associated particle sizes within the nanoscale range, together with the dose-dependent increase in blood Se concentration, provides useful evidence supporting the biological availability of the locally produced Se-enriched yeast. In addition, the study was conducted in Indonesian Thin-Tail sheep under practical tropical production conditions, increasing the relevance of the findings for regional small-ruminant nutrition.

Overall, Se-enriched yeast at 0.35–0.70 g/head/day can be considered a promising dietary Se source for improving systemic Se status without adversely affecting growth, hematological stability, or overall rumen fermentation. The increase in ruminal iso-valerate further indicates a potential nutritional role in supporting microbial nitrogen metabolism. These findings support the use of locally produced Se-enriched yeast as a practical feed supplement for sheep; however, longer-term studies with larger populations are required to confirm its effects on antioxidant capacity, tissue Se deposition, nutrient digestibility, productivity, and long-term safety.

## DATA AVAILABILITY

The data supporting the findings of this study are available within this manuscript.

## GENERATIVE AI DECLARATION

The authors used ChatGPT (OpenAI) solely to improve the English language, grammar, clarity, and readability of the manuscript. The AI tool was not used to generate scientific content, analyze data, interpret results, or draw conclusions. All scientific content was independently developed, verified, and approved by the authors, who take full responsibility for the accuracy, originality, and integrity of the final manuscript

## AUTHORS’ CONTRIBUTIONS

DP: Conceptualization, methodology, validation, investigation, data curation, project administration, and writing – original draft. MMDU: Formal analysis, visualization, validation, writing – original draft, and writing – review and editing. SW: Conceptualization, methodology, supervision, and writing – review and editing. HS: Supervision and writing – review and editing. MA and AHP: Conceptualization, methodology, validation, supervision, and writing – review and editing. All authors have read and approved the final version of the manuscript.
